# Comparison of Clinical Outcomes Between Chinese Patients Receiving Hepatectomy With or Without Enhanced Recovery After Surgery Strategy

**DOI:** 10.3389/fsurg.2021.645935

**Published:** 2021-03-26

**Authors:** Tieli Kang, Zhishuo Jia, Guoquan Xing, Quanhe Zhou

**Affiliations:** Department of Hepatobiliary Surgery, Inner Mongolia Xing'an Meng People's Hospital, Ulanhot, China

**Keywords:** clinical outcome, enhanced recovery after surgery strategy, hepatectomy, Chinese patients, hepatic function

## Abstract

**Purposes:** For the first time in China, the current study was designed to compare the clinical outcomes between Chinese patients receiving hepatectomy with or without the enhanced recovery after surgery (ERAS) strategy.

**Methods:** The current study enrolled 250 patients who would receive hepatectomy. Patients were randomized into two groups: ERAS group (*n* = 125, ERAS strategy) and control (*n* = 125, conventional care). Mortality, length of hospital stay, readmission, and complications were assessed over 30 days after the operation.

**Results:** The average age of the whole cohort was 65 (63–68) years, with 152 males (60.8%). There was no difference between two groups in baseline features, such as age, sex, medical history, Child–Pugh hepatic function, American Society of Anaesthesiologists physical status, operative type, hepatectomy type, and hepatic pathology (*P* > 0.05 for all). There was no occurrence of death in the two groups. Patients in the ERAS group had significantly less occurrence of post-operative complications and a shorter length of hospital stay (*P* < 0.05 for all). Deep vein thrombosis occurred in seven patients in the control group, but did not occur in the ERAS group (*P* < 0.05). Patients in the two groups had similar occurrence of readmission (*P* > 0.05).

**Conclusions:** ERAS strategy significantly decreased the occurrence of operative complications and shortened the length of hospital stay without any increase in mortality or readmission in Chinese patients receiving hepatectomy.

## Background

Enhanced recovery after surgery (ERAS) protocols were first introduced in colorectal operations and have been gradually applied in perioperative management of other diseases (1, 2). ERAS strategy offers a number of advantages, including good guidance and professional management, improved aesthetic and analgesic methods, less use of drainage tubes, and early oral intake and mobilization. It has the chance to not only improve bodily function and accelerate patient recovery, but also decrease the occurrence of operative complications and shorten the length of hospital stay (3).

Hepatectomy is an effective and safe therapy for hepatic tumors (4–6). Previous studies have found that ERAS strategy was beneficial to patients receiving hepatectomy (7–9). However, limited studies have assessed the ERAS strategy in patients receiving hepatectomy in China. More importantly, there is nearly no studies comparing the clinical outcomes of patients receiving hepatectomy with or without ERAS strategy all over the world. For the first time in China, the current study was designed to compare the clinical outcomes between Chinese patients receiving hepatectomy with or without ERAS strategy.

## Methods

### Patients

From January 2011 to December 2017, the current study enrolled 250 patients who would receive hepatectomy at the Department of Hepatobiliary Surgery, Inner Mongolia Xing'an Meng People's Hospital. Inclusion criteria contained the following: (1) partial or half hepatectomy (either right or left lobe); (2) hepatic function: Child-Pugh A or B; (3) physical status: American Society of Anaesthesiologists (ASA) I to III; and (4) no main concomitant operation, such as bowel, gastro, or bile duct resection. Exclusion criteria were the following: (1) unwillingness to participate; (2) hepatic function: Child–Pugh C; (3) physical status: ASA IV or V; or (4) involved the inferior vena cava or confluence of the hepatic vein. The study protocol was approved by the Ethics Committee of Inner Mongolia Xing'an Meng People's Hospital. All participants provided written informed consent. Clinical investigation was conducted according to the principles of the Declaration of Helsinki.

### Procedures

Patients were randomized into two groups, ERAS group (*n* = 125, ERAS strategy) and control (*n* = 125, conventional care), using random numbers in a randomized block design. Random numbers were generated by SPSS 18.0 software (SPSS Inc., Chicago, USA). ERAS strategy was conducted and shown in Table 1. All patients in the ERAS group received ERAS strategy, with an adherence rate of 100%. Hepatectomy was conducted by a united clinical team with full experience. Patients were placed in the supine position, and operations were conducted under general anesthesia.

**Table 1:** ERAS strategy conducted in the current study.

**Strategy**	**Control group**	**ERAS group**
**Pre-operative**		
Pre-operative education	Conventional	Detailed (ERAS strategy)
Oral diet	Nothing for 8 h before operation	Carbohydrate solution (250 ml) 2 h before operation
Bowel preparation	Yes	No
**Operative**		
Patient-controlled analgesia pump	No	Yes
Nasogastric tube	Conventional	Minimized
Room temperature	Conventional	>25°C
Heated liquid	No	Yes
Warm air	No	Yes
Abdominal cavity drainage tube	Conventional	Minimized
Fluid administration	Conventional	Restricted (<2,000 ml)
**Post-operative**		
Urinary catheter removal	3 day after operation	1 day after operation
Abdominal drainage tube removal (<30 ml)	4 day after operation	2 day after operation
Mobilization on bed	2 day after operation	1 day after operation (4 times per day)
Mobilization out of bed	3 day after operation	1 day after operation (4 times per day)
Water	12 h after operation	6 h after operation
Liquid food	2 day after operation	1 day after operation
Semiliquid diet	3 or 4 day after operation	2 day after operation
Normal diet	5 or 6 days after operation	3 day after operation
Fluid administration	Conventional	Restricted (<2,500 ml)

*ERAS, early recovery after surgery*.

### Outcomes

The primary outcomes were mortality over the 30 days after the operation and the length of hospital stay after the operation (the number of days from operation to discharge). The secondary outcomes were assessed by readmission and complications (Clavien–Dindo class) over the 30 days after operation (10). Follow-up was achieved through communicating with patients and their relatives, and readmission was rechecked in the medical records of other hospitals.

### Statistics

Continuous variables with normal distribution were described using mean and standard deviation and compared between two groups using Student's *t*-test. Continuous variables with skewed distribution were described using median and interquartile range and compared between the two groups using Mann-Whitney *U*-test. Categorical variables were described with number and percentage and compared between two groups using Chi-square test. All analyses were conducted using SPSS 18.0 software (SPSS Inc., Chicago, USA). *P* < 0.05 was considered to be statistically significant.

## Results

The average age of the cohort was 65 (63–68) years, with 152 males (60.8%). As shown in Table 2, there was no difference between the two groups in baseline features of patients, such as age, sex, medical history, Child–Pugh hepatic function, ASA physical status, operative type, hepatectomy type, and hepatic pathology (*P* > 0.05 for all). There was no occurrence of death in the two groups. Clinical outcomes of patients were compared between the two groups in Table 3. Patients in the ERAS group had significantly less occurrence of post-operative complications and a shorter length of hospital stay (*P* < 0.05 for all). Deep vein thrombosis (DVT) occurred in seven patients in the control group but did not occur in the ERAS group (*P* < 0.05). No complication in Grade IIIb, IV, or V occurred in the two groups. Patients in the two groups had similar occurrences of readmission (*P* > 0.05).

**Table 2:** Baseline features of all patients divided into ERAS and control groups.

**Features**	**All patients** ** (*n* = 250)**	**Control** ** group** ** (*n* = 125)**	**ERAS** ** group** ** (*n* = 125)**	***P*-value**
Age, years	65 (63–68)	65 (63–68)	65 (63–69)	0.429
Males (%)	152 (60.8)	73 (58.4)	79 (63.2)	0.437
**Medical history (%)**				
Cirrhosis	74 (29.6)	35 (28.0)	39 (31.2)	0.579
Hypertension	99 (39.6)	51 (40.8)	48 (38.4)	0.698
Diabetes mellitus	65 (26.0)	29 (23.2)	36 (28.8)	0.313
Child–Pugh hepatic function (%)				0.776
A	237 (94.8)	119 (95.2)	118 (94.4)	
B	13 (5.2)	6 (4.8)	7 (5.6)	
ASA physical status (%)				0.662
I	41 (16.4)	23 (18.4)	18 (14.4)	
II	185 (74.0)	91 (72.8)	94 (75.2)	
III	25 (10.0)	11 (8.8)	13 (10.4)	
Operative type (%)				0.655
Open hepatectomy		94	97	
Laparoscopic hepatectomy		31	28	
Hepatectomy type (%)				0.541
Left hepatectomy	132 (52.8)	65 (52.0)	67 (53.6)	
Right hepatectomy	89 (35.6)	43 (34.4)	46 (36.8)	
Segmentectomy	29 (11.6)	17 (13.6)	12 (9.6)	
Hepatic pathology (%)				0.370
Hepatocellular carcinoma	167 (66.8)	86 (68.8)	81 (64.8)	
Metastatic hepatic carcinoma	35 (14.0)	15 (12.0)	20 (16.0)	
Cholangiocellular carcinoma	31 (12.4)	13 (10.4)	18 (14.4)	
Hepatic hemangioma	17 (6.8)	11 (8.8)	6 (4.8)	

*ERAS, early recovery after surgery; ASA, American Society of Anesthesiologists*.

**Table 3:** Clinical outcomes of all patients divided into ERAS and control groups.

**Outcomes**	**Control group** ** (*n* = 125)**	**ERAS group** ** (*n* = 125)**	***P*-value**
Length of hospital stay, days	9 (7–11)	8 (6–10)	0.022
Readmission (%)	6 (4.8)	3 (2.4)	0.497
Complication (%)^*^			
No	65 (52.0)	90 (72.0)	0.001
**Grade I**			
Nausea/vomiting	12 (9.6)	8 (6.4)	0.351
Wound infection	6 (4.8)	4 (3.2)	0.519
Deep vein thrombosis	7 (5.6)	0 (0)	0.021
**Grade II**			
Liver failure	16 (12.8)	13 (10.4)	0.554
**Grade IIIa**			
Bile leakage	5 (4.0)	3 (2.4)	0.719
Intraperitoneal inflammation	8 (6.4)	5 (4.0)	0.393
Pleural effusion	6 (4.8)	2 (1.6)	0.281

* *Clavien–Dindo complication class*.

## Discussion

Hepatectomy is a common operation for hepatic tumors (4–6). ERAS strategy has been found in previous studies to significantly shorten the length of hospital stay after hepatectomy (11). Also, research has shown that ERAS strategy decreased the occurrence of operative complications in patients receiving hepatectomy (2). ERAS strategy has the chance to benefit patients receiving hepatectomy (5–9). However, limited studies have assessed the ERAS strategy in patients receiving hepatectomy in China. More importantly, nearly no study has compared the clinical outcomes of patients receiving hepatectomy with or without ERAS strategy all over the world. For the first time in China, ERAS strategy was demonstrated in the current study to have the following effects on patients receiving hepatectomy: (1) reduced occurrence of operative complications, (2) shortened length of hospital stay, and (3) no increased mortality or readmission. The advantages of the ERAS strategy, such as good guidance and professional management, improved anesthetic and analgesic methods, less use of drainage tubes, and early oral intake and mobilization, could be beneficial in accelerating the function recovery and achieving better outcomes.

The current study confirmed that the ERAS group had a significantly lower occurrence of complications, and identified DVT as a major complication prevented by ERAS strategy. Limited fluid therapy and early oral diet have been reported to not only promote the resumption of bowel function and lessen the stress reaction of patients, but also decrease post-operative complications and shorten hospitalization time (12). Meanwhile, good pain control has also been recommended to achieve rapid recovery and discharge and decrease the occurrence of post-operative complications (2).

Good guidance and management could be significant to the smooth implementation of the ERAS strategy. Before hepatectomy, it is essential for patients to understand the ERAS strategy and follow the advice of medical management (2). Unhealthy emotions in patients occur frequently and cannot be ignored in clinical practice (13). These emotions can prolong the length of hospital stay and increase the occurrence of operative complications (13). ERAS strategy provides a series of effective ways to shift the patients' insecure concerns and relieve their worries and stress (14). Moreover, good communication and cooperation could further avoid these unhealthy emotions. After hepatectomy, professional guidance and management from a united clinical team guarantees meeting the daily goals and ensures the effective implementation of the ERAS strategy (15). Each point of the ERAS strategy should be helpful for effective improvement of function and rapid recovery of patients (16). A professional team means all patients will achieve the goals of ERAS strategy, and good communication increases the involvement of patients in decisions and the therapeutic process (17).

Less use and early removal of tubes could be a feasible way to decrease the occurrence of complications and shorten the length of hospital stays. During hepatectomy, nasogastric tubes are temporarily used only under conditions of stomach gas and are removed at the end of the operation (18). After hepatectomy, less use and early removal of tubes promote early mobilization and decrease post-operative complications (19). Patients have healthy emotions and early mobilization as they suffer from fewer tubes and less pain (20, 21). With the help of post-operative monitoring, patients with less use and early removal of drainage tubes tend to have early mobilization and recovery, but not increased mortality or readmission.

The current study had some limitations. Firstly, the current study had a relatively small number of patients, and larger studies are essential for the future. Secondly, the current study enrolled all patients from January 2011 to December 2017, and then only had a short-term period of follow-up. Long-term follow-up is being conducted and will be presented in the future.

## Conclusions

For the first time in China, the current study demonstrated that ERAS strategy significantly decreased the occurrence of operative complications and shortened the length of hospital stay without any increase in mortality or readmission in Chinese patients receiving hepatectomy.

## Data Availability Statement

The original contributions generated in the study are included in the article/supplementary material, further inquiries can be directed to the corresponding author.

## Ethics Statement

The studies involving human participants were reviewed and approved by Ethics Committee of Inner Mongolia Xing'an Meng People's Hospital. The patients/participants provided their written informed consent to participate in this study.

## Author Contributions

All authors conceived and designed the experiments, performed the experiments, analyzed the data, contributed reagents, materials, analysis tools, and wrote the paper.

## Conflict of Interest

The authors declare that the research was conducted in the absence of any commercial or financial relationships that could be construed as a potential conflict of interest.
